# Editorial for Special Issue: “Production and Role of Molecular Hydrogen in Plants”

**DOI:** 10.3390/plants11152047

**Published:** 2022-08-05

**Authors:** John T. Hancock

**Affiliations:** Department of Applied Sciences, University of the West of England, Bristol BS16 1QY, UK; john.hancock@uwe.ac.uk; Tel.: +44-(0)1173282475

## 1. Introduction

Molecular hydrogen (H_2_) is an extremely small molecule, which is relatively insoluble in water and relatively inert. Regardless of this, there seems little doubt that H_2_ has profound effects in a range of organisms, from plants [1] to humans. In the biomedical arena, H_2_ has been suggested as a therapeutic agent [2] for a range of diseases, including neurodegenerative disease [3] and COVID-19 [4]. It has even been suggested to be useful as a sports supplement [5].

H_2_ can be administered to plants, or plant tissues, either as a gas or dissolved in a suitable medium and sprayed on. For the latter, H_2_ is usually bubbled through water to make a hydrogen-enriched solution, referred to as hydrogen-rich water (HRW). This can then be added to the soil (or feed solution) or sprayed on the foliage. As an example, Wu et al. [6] used this approach to look at effects of H_2_ on cadmium stress in cabbage.

Using such approaches there have been numerous reports of the effects of H_2_ in plants. H_2_ is involved in seed germination, for example, especially under salt stress [7]. Hydrogen-rich water (HRW) promotes root growth, again especially under stress conditions [8], such as when excess metal ions are present. It has been suggested that such stress relief by H_2_ may involve phytohormone signalling [9].

H_2_ is safe. Sun et al. [10] give three reasons why they consider H_2_ safe for humans, and, therefore, by extension it is safe for treatments of plants, which are used for food crops. Firstly, H_2_ has been used as a compressed gas for deep-sea diving for decades, with no ill effects reported. Secondly, H_2_ is an endogenous gas, being produced in the gut, for example [11]. Thirdly, experimental evidence has been reported that H_2_ is safe. On the other hand, H_2_ is highly inflammable, so some caution needs to be exercised if used in confined places, such as in a glasshouse.

How H_2_ is acting on plants is not well understood. If sprayed onto the foliage it is not known how much of the H_2_ enters the plant tissues but clearly it has to if there are effects seen. Even if used as HRW, H_2_ is very likely to enter the gas phase relatively quickly, so repeated treatments may be needed. The direct effects of H_2_ are also hard to understand, i.e., what the molecular targets of H_2_ are. H_2_ is probably too small to interact with a protein receptor in the classical manner. However, there are reports of H_2_ acting as an antioxidant [12], with the most likely target being the hydroxyl radical, or the peroxynitrite molecule (ONOO^−^). The latter is formed through a reaction of nitric oxide (NO) and superoxide anions. It is thought that it is less likely that H_2_ is reacting with other reactive oxygen species (ROS), such as the superoxide anion or hydrogen peroxide (H_2_O_2_), or other reactive nitrogen species, such as NO. It is also unlikely that H_2_ is involved in the direct modification of amino acids in polypeptides, which would be the typical mechanism of H_2_O_2_ (through oxidation [13]) or of NO (through *S*-nitrosylation [14]). Others have suggested that H_2_ acts on enzymes, such as heme oxygenase [15], but the direct interaction is not known.

Here, this Special Issue (SI) was an invitation to those in the field to give their up-to-date research and appraisal of the effects and uses of H_2_ in plant science.

## 2. Effects of H_2_ on Plants

Here, in this SI, Nguyen and Lim ask if H_2_ can be used for the extension of the vase life of flowers [16]. Post-harvest is a hugely important topic. In some countries, the postharvest waste has been estimated to be over 40% [17]. Crops need to be commercially viable, safe to consume and acceptable to the customers. This latter point is very pertinent for flowers where the expectation would be that they look good and last for as long as possible. Nguyen and Lim reviewed the various methods used to deliver H_2_ to plants, including HRW, hydrogen nanobubble water (HNW) and magnesium hydride (MgH_2_). Plants used across studies included rose, carnation and lilies. Overall, the application of H_2_ increased vase life of flowers, and the authors concluded that such work should be continued to release the potential for the use of H_2_ in the floriculture industry. Interestingly, the authors also discuss the cost–benefit analysis of H_2_ use and suggest that labour costs are an issue [18]. Certainly, as H_2_ is developed more for other industries, such as a transport energy source [19], the cost and delivery of H_2_ is likely to become cheaper.

Li et al. [20] continue the theme of using H_2_ to extend the vase life of flowers. Here, the authors use HNW, which they say has a higher concentration of dissolved H_2_ than conventional HRW, and the residual time that the H_2_ remains dissolved for is longer, both properties, which would be of benefit if adopted for widespread use. Their data show that 5% HNW significantly lengthened the vase life of cut carnations. This concentration was better than other concentrations of HNW tried, and better than either water or HRW. Their measures of improvements included electrolyte leakage, oxidative damage and cell death in the petals. The authors concluded by suggesting that HNW may have future applications for postharvest preservation. Certainly, treatment with molecular hydrogen is likely to be much safer than the use of some of the alternatives mooted, such as hydrogen sulfide [21], which is known to have toxicity [22]. This might not be too much of an issue with flowers but may become a problem if the same treatments are used for postharvest preservation of food crops.

Cheng et al. [23] have taken H_2_ applications out into the field, with trials of whether such treatments will improve rice. They too use HNW, comparing it to ditch water. Their data show that HNW increased the length, width and thickness of brown/rough rice and white rice. They then looked at gene expression in these plants and could correlate the physiological changes with the molecular alterations seen. In the white rice they saw no difference in total starch content, but the enzyme amylase was decreased. Cadmium accumulation was also decreased, which also correlated with gene expression patterns reported. Overall, such work shows that H_2_ treatments can be taken to larger scales, not just in a laboratory setting. The authors conclude that H_2_ application does increase the quality of the rice and should be considered as a future treatment. Although field trials with H_2_ have been written about before [24], they are relatively rare and more large-scale work, such as this, certainly needs to be undertaken if H_2_ treatments are to be used more widely in agriculture.

There seems little doubt that H_2_ treatment of plants is beneficial, as exemplified by the papers in this SI and the further papers that these authors cite. There is a growing body of this evidence, and as more is reported, on different species, the use of H_2_ will be seen to be advantageous, whether used in the field or postharvest. Despite the molecular mechanisms not being well understood, the next papers go someway to unravel what H_2_ might be doing in plant tissues.

## 3. Mechanisms of H_2_ Action

The mechanisms in the cells, which enable H_2_ to have its effects, were also the focus of papers in this SI. Zhao et al. [25] show an interaction between H_2_ and glucose in adventitious roots of cucumber. The effects of HRW were blocked by glucosamine, suggesting that glucose content may be mediating root development. HRW increased the cellular content of a range of sugar-based metabolites, including glucose, starch, sucrose, glucose-6-phosphate, fructose-6-phosphate and glucose-1-phosphate. HRW treatment resulted in the increase in the activity of several relevant enzymes, including hexokinase, pyruvate kinase and sucrose synthase, and, furthermore, gene expression patterns matched these findings. Interestingly, the authors state that all the positive effects of HRW were inhibited by glucosamine, and they concluded that H_2_ was regulating adventitious root growth by promoting glucose metabolism.

The literature on H_2_ effects tends to support the notion that H_2_ increases cellular antioxidant levels. For example, Wu et al. [6] reported increased antioxidants in cabbage under cadmium stress, whilst Chen et al. [26] showed that the antioxidant capacity of *Hypsizygus marmoreus* (mushroom) was increased by HRW use during postharvest. Here, in this SI, Jiang et al. [27] also look at antioxidant capacity and how this might be improved by H_2_ treatment, using Chinese chive. In a similar manner to the vase-life work above, this is also being carried out postharvest. Chives were treated with a range of H_2_ concentrations, in comparison to air. Shelf-life was improved most by 3% H_2_, a conclusion supported by measurements of decay index, loss ratio of weight and protein content. Of pertinence to the discussion here, the content of total phenolics, flavonoids and vitamin C were maintained, whilst the activities of antioxidant enzymes were increased, including superoxide dismutase (SOD), catalase (CAT) and ascorbate peroxidase (APX). Clearly, H_2_ treatment was protecting the plant material by an increase in antioxidant capacity, as suggested by others, for example [28].

Zhang et al. [29] looked at pesticide residues in plants. Using tomato and Arabidopsis, they found that the degradation of carbendazim (a benzimidazole pesticide) was increased by H_2_. H_2_ increased glutathione metabolism, which led to the increased degradation of carbendazim. Glutathione is an immensely important antioxidant in plants [30], so any effects seen may also alter cellular redox states [31]. Carbendazim is a fungicide [32]. Zhang et al. [29] comment that the antifungal action of carbendazim was not affected by the H_2_ treatments, but they were convinced that glutathione was important for the H_2_ detoxification of the fungicide. Therefore, as the authors point out, this is a previously unknown action of H_2_ in plant cells.

Wuzhimaotao (*Ficus hirta Vahl*) was the plant of focus for the study by Zeng and Yu [33]. In China, this edible plant also has medicinal properties. Following treatment with H_2_ (as HRW), the transcriptomic pattern of the roots was compared with controls. One hundred and seventy three genes were found to be down regulated, whilst 138 were up regulated. The authors also carried out a metabolomic analysis and found that nearly 200 metabolites had their levels altered by H_2_ treatment. With further analysis, it was suggested that the biosynthesis and metabolism of phenylpropanoid were the main pathways being controlled by H_2_ treatment. Although the authors suggest that H_2_ application should be considered for future growth of this medicinal plant, the data also show the scale of the effects that can be reported when plants are treated with H_2_. With so many genes being up- and down-regulated, H_2_ must be having a profound effect on transcription factors in plant cells. With similar results emanating from work on mammalian cells [34], the future will no doubt allow the mechanisms behind these changes in gene expression to be unravelled.

Finally, a review of the direct actions of H_2_ on plants was published as part of the SI [35]. There are reports that H_2_ acts as a direct antioxidant [12], but the chemistry has been disputed [36]. It has been suggested that H_2_ acts through its redox state [37], and there is some precedent for this in bacterial systems [38]. Alternatively, H_2_ may be acting through its spin states [39], but there is no evidence of this. Clearly, there is a lot to explore here, and some of the focus of H_2_ research needs to be pointed in this direction.

Whatever the mechanism, it would strengthen the argument for the use of H_2_ in agriculture, and in the biomedical arena, if the mechanism(s) of the direct action of H_2_ was resolved, but there is no doubt that such evidence, either ruling mechanisms in or out, will be forthcoming in the future.

## 4. Conclusions and Future Perspectives

As can be seen by the papers that were published in this Special Issue, H_2_ has beneficial effects on plants. H_2_ appears to increase the crop yields and can be used for improving postharvest storage of crops, as exemplified by the work on flowers here. Therefore, H_2_ use should have a bright future in agricultural settings.

Plants may be exposed to H_2_ naturally, either through the action of cellular enzymes [40], or through the metabolism of other organisms in the location, such as in the soil [41]. Alternatively, H_2_ may be applied to the plant—either onto the soil or foliage—as a treatment. As can be seen in the papers in this SI, there are a variety of ways to achieve this. H_2_ can be applied as a gas, or in an enriched solution, i.e., HRW. However, more recent advances in this area have seen the development of other solutions, which can be used, such as HNW. Alternatively, H_2_ can be supplied from donor molecules, such as MgH_2_, and no doubt the future will see better donor compounds being developed, which can deliver more H_2_ for a longer period of time, rather than giving tissues a bolus effect.

One aspect of the reporting of the effects of H_2_ that needs to be consistent is the quoting of the concentration of H_2_ used. Often the percentage of HNW or HRW is quoted, but without knowing for sure exactly how concentrated the stock solution is it is hard to compare different studies and, therefore, the effects. H_2_ does not last long in solution, so quoting the actual concentration of H_2_ in the solutions used would be very beneficial to push this field forward.

Some evidence of the molecular aspects are presented here in this SI too, including the action through glucose metabolism, glutathione metabolism, as an antioxidant and in the control of gene expression. However, the direct targets of H_2_ still remain elusive, not just in plant science but in all aspects of the action of H_2_ in biological systems. Several ideas have been mooted but there is little evidence of them at present. Future work needs to be focussed on this aspect of H_2_ biology, as this would really strengthen the argument for H_2_ use. It would also give reassurance on the safety of H_2_ treatments, especially if it is proposed to be used as a treatment for consumed crops, either in the field or postharvest.

Finally, more large field trials are needed on a range of crops. H_2_ is being studied in some countries around the world, most notably China, but it needs to be looked at more widely, in different locations and with different plants. H_2_ appears to be safe, albeit inflammable, but the cost–benefit needs to be well established before H_2_ will be taken up widely in agriculture and floriculture. H_2_ has benefits, especially if used when plants are stressed, and no doubt large scale trials will unlock the hesitation for the adoption of H_2_ applications in the future.
